# Altered static and dynamic spontaneous neural activity in patients with ischemic pontine stroke

**DOI:** 10.3389/fnins.2023.1131062

**Published:** 2023-03-16

**Authors:** Xin Wang, Caihong Wang, Jingchun Liu, Jun Guo, Peifang Miao, Ying Wei, Yingying Wang, Zhen Li, Jie Li, Kaiyu Wang, Yong Zhang, Jingliang Cheng, Cuiping Ren

**Affiliations:** ^1^Key Laboratory for Functional Magnetic Resonance Imaging and Molecular Imaging of Henan Province, Department of MRI, The First Affiliated Hospital of Zhengzhou University, Zhengzhou, China; ^2^Tianjin Key Laboratory of Functional Imaging, Department of Radiology, Tianjin Medical University General Hospital, Tianjin, China; ^3^Department of Radiology, Tianjin Huanhu Hospital, Tianjin, China; ^4^Department of Interventional Radiology, The First Affiliated Hospital of Zhengzhou University, Zhengzhou, China; ^5^GE Healthcare MR Research, Beijing, China

**Keywords:** dynamic intrinsic brain activity, pontine infarction, regional homogeneity, amplitude of low-frequency fluctuations, functional magnetic resonance imaging

## Abstract

**Objective:**

The purpose of the study was to investigate the abnormality both of static spontaneous brain activity and dynamic temporal variances following a pontine infarction.

**Methods:**

Forty-six patients with chronic left pontine infarction (LPI), thirty-two patients with chronic right pontine infarction (RPI), and fifty healthy controls (HCs) were recruited for the study. The static amplitude of low-frequency fluctuations (sALFF), static regional homogeneity (sReHo), dynamic ALFF (dALFF), and dynamic ReHo (dReHo) were employed to detect the alterations in brain activity induced by an infarction. The Rey Auditory Verbal Learning Test and Flanker task were used to evaluate the verbal memory and visual attention function, respectively. Receiver operating characteristic curve analysis was used to reveal the potential capacity of these metrics to distinguish the patients from HCs.

**Results:**

There were significant variations of these static and dynamic metrics in patients with chronic pontine infarction. The altered regions involved the supratentorial regions, including cortex and subcortical. Moreover, the altered metrics were significantly correlated with verbal memory and visual attention. In addition, these static and dynamic metrics also showed potential in distinguishing stroke patients with behavior deficits from HCs.

**Conclusion:**

The pontine infarction-induced cerebral activation changes are observed in both motor and cognitive systems, indicating the functional damage and reorganization across the global cerebral level in these patients with subtentorial infarction, and there is a reciprocal effect between motor and cognitive impairment and repair.

## Introduction

Pontine infarction (PI) is caused by occlusion of the main basilar artery and its branches (Kim and Caplan, 2016). This commonly results in complex and varied symptoms, such as motor and sensory disorders, peripheral facial paralysis, and ataxia of the affected side (Bassetti et al., 1996; Oh et al., 2015). In addition, prior studies indicated that PI may also lead to cognitive dysfunction (Obayashi, 2019; Wu et al., 2021). It may be that a brainstem lesion caused damage to the direct or indirect anatomical pathways between brainstem and regions of the cortex. However, PI-induced cognitive deficits tend to manifest delayed and may be easily overlooked. This may influence the long-term prognosis and quality of life of these patients, as well as increase the risk of recurrent stroke. Therefore, exploring the characteristics of cognitive impairment caused by PI is helpful to guide the long-term rehabilitation of patients and improve their prognosis.

Resting-state functional magnetic resonance imaging (rs-fMRI) is a promising tool for investigating spontaneous brain activity by measuring low-frequency fluctuations in blood oxygen level-dependent (BOLD) signals (Wang et al., 2020). Brain disorders can disturb spontaneous neuronal activity, resulting in an imbalance in energy metabolism (Fox and Raichle, 2007). Numerous studies have demonstrated that low-frequency fluctuations are critical for comprehending human brain activity under both normal and abnormal conditions (Auer, 2008; Lee et al., 2013). The static amplitude of low-frequency fluctuations (sALFF) and regional homogeneity (sReHo) are two primary quantitative metrics typically used to measure the activity of regional low-frequency spontaneous neurons (Shen et al., 2020; Huang et al., 2021). The two approaches which have been effectively used to detect abnormal neuronal activity in neuropsychiatric disorders, such as Attention deficit and hyperactivity disorder (Wang et al., 2013), Alzheimer’s disease (Zhang et al., 2012), and Parkinson’s disease (Yue et al., 2020). The sALFF could reflect the strength of intrinsic brain activity by measuring the spontaneous neural activity in a local cerebral region within the range of 0.01 and 0.1 Hz (Tsai et al., 2014b). Tsai et al. (2014a) discovered that there were significant differences in ALFF values across the infarct core tissue, penumbra tissue, and healthy brain tissue. This indicates that the ALFF may provide potential value in distinguishing between normal and pathological brain regions. In addition, the previous studies showed that there were significantly increased ALFF in motor cortical areas (i.e., supplementary motor area) and cognitive cortical areas (i.e., hippocampus, parahippocampus), and significantly decreased ALFF in the cerebellum in stroke patients (Tsai et al., 2014b; Xie et al., 2022). Altered ALFF can also be detected in chronic stroke patients before and after treatment intervention (Hu et al., 2018). The sReHo indicates the similarity of local neural activity among adjacent regions, which is measured by Kendall coefficients. Studies showed that patients with subcortical stroke displayed significantly altered sReHo in cortical (Wu et al., 2015) and subcortical (Li et al., 2022) regions. Patients with pontine stroke displayed significantly altered sReHo in cortical regions (Jiang et al., 2019), as well. The findings of these studies suggest that these two quantitative metrics may be important neurophysiological indicators for exploring intrinsic brain activity in stroke patients.

Most of the aforementioned studies investigated the regional brain activity under the presumption that the BOLD signal was unchanged during the fMRI scanning. However, it neglects the fact that local cerebral activity has dynamic properties across a time-varying process (Liu and Duyn, 2013; Zalesky et al., 2014; Cohen, 2018). The utility of dynamic measures has a significant advantage over static ones in monitoring recurring brain activity (Xie et al., 2018). Dynamic research may complement the deficiencies of static alteration, and their combination may provide a more comprehensive explanation of neuropathological changes in chronic stroke. The sliding window is the main method of dynamic analysis techniques and has been widely utilized in the temporal variability of aberrant brain function in neuropsychiatric disorders (Cui et al., 2020; Liu et al., 2021; Zhao et al., 2021). The dynamic ALFF (dALFF) and dynamic ReHo (dReHo) are two commonly used analysis techniques for analyzing dynamic brain activity. The dynamic local methods of dALFF and dReHo based on time-variant brain activity explore the variability and regional synchronization in the amplitude of oscillations of spontaneous brain activity in brain injuries and cognitive disorders. This could potentially improve our understanding of the neuromechanism of brain activity by recognizing specific pathophysiological features associated with behavioral disorders. It has been found that there were significantly altered dALFF and dReHo in multiple cortical regions in subacute stroke patients, which were related to motor function (Chen et al., 2018). However, numerous studies mainly focus on supratentorial stroke patients, while little is known about the dynamic change characteristics in patients with subtentorial chronic stroke (Liu et al., 2017; Tian et al., 2022).

In the current study, we integrated static (sALFF, sReHo) and dynamic multiple indicators to explore the characteristics of brain activity changes in patients with chronic pontine infarction. The purpose of this study are as follows: (1) to investigate the altered pattern of static intrinsic brain activity and dynamic temporal variances following the pontine infarction, (2) to explore the changes of cognitive function in these patients, and (3) to uncover the relationships between these metrics variability and the alterations of cognitive function in pontine infarction patients.

## Materials and methods

### Subjects

The G* Power software (version 3.1) was used for sample size estimation. Parameters for the test are as follows: two tails, effect size *d* = 0.8, alpha error prob = 0.05, power = 0.90, allocation ratio = 1. Then the sample size of each group is 28. In the study, a total of 78 right-handed patients with unilateral pontine infarction were recruited from three hospitals (The First Affiliated Hospital of Zhengzhou University, Tianjin Medical University General Hospital, and Tianjin Huanhu Hospital). Among them, 46 patients with left pontine infarction (LPI group), and 32 patients with right pontine infarction (RPI group). Additionally, 50 right-handed healthy controls (HCs) were recruited from local communities. One patient with right pontine ischemic was excluded due to large head motion (mean FD > 0.5). Three patients with inappropriate lesion location and one patient with cavum septi pellucidi were excluded from the LPI group, The flowchart for patient selection is shown in Supplementary Figure 1. Finally, 42 LPI patients, 31 RPI patients, and 50 HCs were included in this research. The study was approved by the medical research ethics committees of the three aforementioned hospitals, and the Chinese Clinical Trial Registry (ChiCTR1900027064). All participants signed informed consent forms.

To guarantee data quality, we created a stringent protocol when amalgamating the data from the three centers, which may have considerable variation. The inclusion criteria were as follows: (1) first-onset infarction that involves the left or right pontine; (2) chronic-stage of the infarction (after initial onset >6 months); (3) right-handedness before infarction onset; (4) 40–80 years of age. The exclusion criteria were as follows: (1) recurrent or hemorrhagic infarction; (2) white matter demyelination with a Fazekas scale score >1 (Fazekas et al., 1987); (3) any other serious brain abnormalities; (4) history of drug addiction or neuropsychological illnesses; (5) serious underlying diseases including cardiac arrest and tumor; (6) contraindications for MRI.

### Behavioral evaluation

In the present study, Ray Auditory Verbal Learning Test (RAVLT) was utilized to score the verbal memory function, which is a measure involving both verbal short-term memory (VSTM), and verbal long-term memory (VLTM) (Ricci et al., 2022). The modified version of Flanker task was used to access the visual attention (Haciahmet et al., 2021) based on E-Prime 2.0 software.^1^ The mean reaction time (RT) and accuracy (ACC) were calculated as indicators of visual attention for each participant in the task.

### Data acquisition

The MRI data for the study were acquired using three 3.0-Tesla MR imaging scanners. Two of the scanners were Discovery MR 750 (GE Medical Systems, Waukesha, WI, United States) from The First Affiliated Hospital of Zhengzhou University and Tianjin Medical University General Hospital. The other scanner was a Magnetom Trio Tim MR (Siemens, Erlangen, Germany) from the Tianjin Huanhu Hospital. Participants were instructed to keep their eyes closed and refrain from thinking about anything during the scanning process. To minimize head movements and scanner noise, the subjects wore comfortable foam padding and earplugs.

The images of subjects were collected on the two Discovery MR750 scanners using the same parameters to reduce the scanners variability as much as possible. These are as follows: resting-state functional MRI (fMRI) data were obtained using a gradient echo single-shot echo-planar imaging sequence: TR/TE = 2,000/41 ms; field of view = 220 mm × 220 mm; matrix = 64 × 64; flip angle = 90°; slice thickness = 4 mm; 0.5 mm gap; 32 slices; 180 time points. Anatomical images were acquired using a 3D T1-weighted sequence: TR/TE = 8.2/3.2 ms; FOV = 256 mm × 256 mm; matrix = 256 mm × 256 mm; slice thickness = 1.0 mm, no gap; 188 slices. The Trion Tim parameter was as follows: resting-state fMRI data: TR/TE = 2,000/30 ms, flip angle = 90°, FOV = 220 mm × 220 mm, matrix = 64 mm × 64 mm, thickness = 4 mm, no gap, slices = 36, volumes = 180. 3D-T1WI were acquired using the magnetization prepared rapid acquisition gradient echo (MPRAGE) sequence with the following parameters: TR/TE = 2,000/2.26 ms, flip angle = 9^°^, FOV = 256 mm × 232 mm, matrix = 256 × 232, thickness = 1 mm, and slices = 192, resulting in a 1 mm isotropic voxel.

### Data pre-processing

Resting-state functional MRI (rs-fMRI) data were preprocessed using the DPABI software (Yan et al., 2016)^2^ in the MATLAB environment. The preprocessing steps were as follows: (1) convert images in DICOM to the NIFTI format; (2) removal of each subject’s initial 10 volumes to enable the signal to equalize and the participants to get used to the scanning noise; (3) time correction for the time delay of the remaining 170 volumes between slices; (4) realignment correction and mean framewise displacement (mean FD) were calculated for head motion. None of the subjects exceeded 2 mm translation and 2 rotation, and one patient in the RPI group was removed because of an FD > 0.5; (5) co-registration of the rs-fMRI and 3D T1WI for each subject; (6) then, functional images were spatially normalized to the standard Montreal Neurological Institute (MNI) space based on Diffeomorphic Anatomical Registration Through Exponentiated Lie algebra (DARTEL), and re-sampled into a size of 3 mm^3^ voxels; (7) cerebrospinal fluid signal, white matter, and the Friston 24-parameter head motion model were regressed out as nuisance factors (Friston et al., 1996); (8) a temporal band-pass frequency filter (0.01–0.1 Hz) was applied to eliminate low-frequency drift and high-frequency noise; (9) lastly, the images were smoothed using an 8 mm full width half maximum (FWHM) Gaussian kernel for sALFF calculation.

### sALFF and sReHo calculation

The sALFF and sReHo were calculated using the DPABI software. Every preprocessed time series underwent a fast Fourier transformation to convert it into the frequency domain for the sALFF calculation. The power spectrum’s square root was computed in each subject, and the average square root across the frequency range of 0.01–0.1 Hz was used to get the sALFF value. In addition, the data were then *z*-transformed for the next statistical analyses. Every analysis was carried out on a whole-brain level.

The Kendall’s coefficient of concordance (KCC) was utilized to compute sReHo by the DPABI software. This serves as a measure of the similarity between the time series of a given voxel and its nearest 26 voxels (Zang et al., 2004). Then, the images were *z*-transformed and spatial smoothing with an 8 mm FWHM was performed for the statistical analyses.

### Dynamic ALFF and dynamic ReHo calculation

Analysis of dynamic local metrics (dALFF or dReHo) was carried out utilizing DPABI-based Temporal Dynamic Analysis toolkits (Yan et al., 2016). In this study, the sliding window method was used to calculate the dynamic metrics. This method is effective in detecting temporal changes and assessing the variability of metrics across the brain (Hindriks et al., 2016; Yip et al., 2017; Vergara et al., 2019). The window length used in resting-state dynamic analyses is a key factor. It is used to strike a compromise between detecting quickly changing dynamic spatial characteristics and providing credible evaluations of regional brain activity. The sliding window connectivity has been used in previous studies with lengths of time ranging from 10 to 180 s (Thompson et al., 2013; Gonzalez-Castillo et al., 2015; Chen et al., 2018). Here we used a sliding window length of 30 TRs (60 s) and 141 windows were calculated in the analysis. Furthermore, the window lengths of 20 TRs (40 s) and 40 TRs (80 s) were also calculated for the validation analysis. The standard deviation (SD) of dALFF and dReHo values for all voxels in 141 time windows of each participant was calculated to assess the variability of ALFF and ReHo. Then, the individual map was standardized by dividing the mean dALFF/dReHo of the entire brain to reduce inter-subject variations. Finally, dALFF and dReHo maps were spatially smoothed with an isotropic Gaussian kernel of 8 mm FWHM.

### Statistical analysis

Two-sample *t*-tests or chi-square tests were used in the SPSS 21.0 software (SPSS, Inc., Chicago, IL, United States) to explore group differences in demographic information between the PI and HCs. The Shapiro-Wilk test was used to determine the normality of clinical data. General linear model (GLM) in SPM12 was used to compare static metrics (sALFF, sReHo) and dynamic metrics (dALFF, dReHo) of localized activity in the whole brain between patients with PI and HCs, with age, sex, education, mean FD, and scanners variables as covariates. Multiple comparison correction was performed based on Gaussian random field theory (GRF, voxel-wise *p* < 0.001, cluster-wise *p* < 0.05). Moreover, in order to identify the potential clinical significance of these static and dynamic metrics, the group-level brain regions that showed significant inter-group differences of these metrics were extracted for the receiver operating characteristic (ROC) curve analyses utilizing Graphpad Prism 5.0 software. It was conducted with a significant threshold of *p* < 0.05.

### Correlation between fMRI parameters and clinical function

Extracting the mean values of static (sALFF, sReHo) and dynamic (dALFF, dReHo) metrics from the areas exhibiting significant alteration for the correlation analyses. and comparing these values with RAVLT scores and Flanker-RT to explore their relationships with symptom intensity. RAVLT scores are normally distributed according to the Shapiro-Wilk test, correlations were calculated using Pearson correlation coefficient. Flanker-RT does not follow a normal distribution, correlations were calculated using Spearman correlation coefficient. *P* < 0.05 were considered as statistically significant.

## Results

### Comparisons of demographic and behavioral measures

**FIGURE 1:**
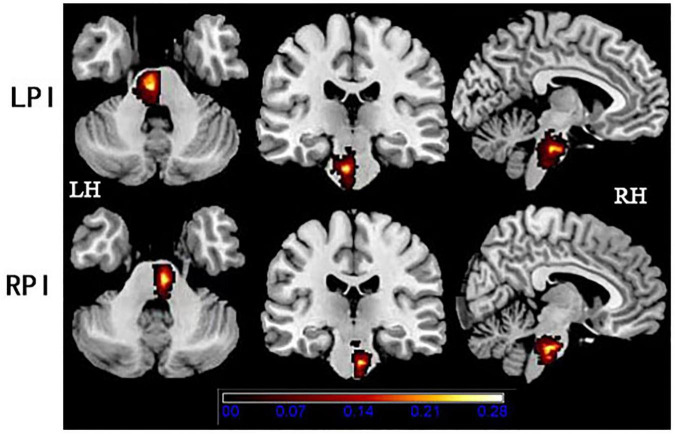
Lesion distribution in the LPI and RPI groups. The color bar represents the lesion probability. LH, left hemisphere; LPI, left pontine infarction; RH, right hemisphere; RPI, right pontine infarction.

Demographic and clinical data are described in Table 1. There were no significant differences in age (*P*_*LPI*_ = 0.29, *P*_*RPI*_ = 0.36), and years of education (*P*_*LPI*_ = 0.69, *P*_*RPI*_ = 0.28) both in the LPI and RPI groups when compared to the HCs group. There were no significant differences in sex between the RPI group (*P*_*RPI*_ = 0.48) and HCs group, but there was a significant difference in the LPI group (*P*_*LPI*_ = 0.005). Lesion size at the chronic phase (*P* = 0.66) and interval time between the stroke onset and the MRI scan (*P* = 0.13) showed no significant differences between the LPI and RPI groups. Compared with HCs group, there were no significant differences in the scores of VSTM (*P*_*LPI*_ = 0.10, *P*_*RPI*_ = 0.58), VLTM (*P*_*LPI*_ = 0.46, *P*_*RPI*_ = 0.15), Flanker-RT (*P*_*LPI*_ = 0.37, *P*_*RPI*_ = 0.74) both in the LPI and RPI group. Furthermore, a significant difference between RPI group and HCs group in mean FD values (*P*_*RPI*_ = 0.02) was found. The lesion distribution map of the stroke patients is shown in Figure 1.

**TABLE 1:** Demographic and clinical data of participants.

Variable	PI (*n* = 73)		HCs (*n* = 50)	*P*-value
	**LPI (*n* = 42)**	**RPI (*n* = 31)**		
Age (years)	56.5 ± 7.44	56.48 ± 8.18	54.82 ± 7.69	LPI = 0.29; RPI = 0.36
Gender (M/F)	33/9	18/13	25/25	LPI = 0.005*; RPI = 0.48
Year of education	9∼(9 12)	9∼(9 12)	10.5∼(9 12)	LPI = 0.69; RPI = 0.28
Lesion size (cc)	0.27∼(0.15 0.44)	0.22∼(0.14 0.41)	–	*P* = 0.66
Mean FD	0.14∼(0.10 0.22)	0.17∼(0.11 0.24)	0.12∼(0.09 0.18)	LPI = 0.20; RPI = 0.02*
Scan time interval after stroke (months)	11∼(6 12)	12∼(10 12)	–	*P* = 0.13
VSTM scores	44.74 ± 11.90	46.86 ± 13.01	48.38 ± 9.18	LPI = 0.10; RPI = 0.58
VLTM scores	10.40 ± 2.96	9.83 ± 3.25	10.86 ± 2.91	LPI = 0.46; RPI = 0.15
Flanker-RT	575.12∼(506.23 645.60)	576.82∼(513.00 748.71)	543.57∼(463.64 703.23)	LPI = 0.37; RPI = 0.74

Data with normal distribution were present with mean ± SD. Data with non-normal distribution were present with P_50_ (P_25_∼P_75_). HCs, healthy controls; LPI, left pontine infarction; RPI, right pontine infarction; RT, mean reaction time. VLTM, verbal long-term memory of Ray Auditory Verbal Learning Test (RAVLT); VSTM, verbal short-term memory of Ray Auditory Verbal Learning Test (RAVLT); *P < 0.05.

### Differences in sALFF and dALFF

**FIGURE 2:**
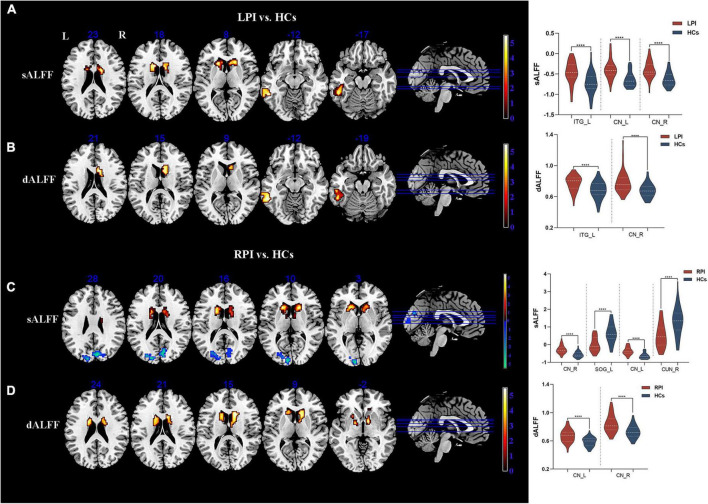
sALFF/dALFF differences between pontine infarction (PI) and healthy controls (HCs) group. **(A)** Voxel-based analysis showed brain regions with significant sALFF alterations of LPI group. **(B)** Voxel-based analysis showed brain regions with significant dALFF alterations of LPI group. **(C)** Voxel-based analysis showed brain regions with significant sALFF alterations of RPI group. **(D)** Voxel-based analysis showed brain regions with significant dALFF alterations of RPI group. dALFF, dynamic amplitude of low-frequency fluctuations; LPI, left pontine infarction; RPI, right pontine infarction; sALFF, static amplitude of low-frequency fluctuations; ****Represents *p*-value < 0.0001.

The significant differences in sALFF and dALFF (the window length of 30 TRs) were shown in Figure 2, Table 2. The verification analysis results of the two additional window lengths (20 TRs and 40 TRs) were displayed in Supplementary Figures 2, 3, Supplementary Tables 2, 3.

**TABLE 2:** Static [static amplitude of low-frequency fluctuations (sALFF), static regional homogeneity (sReHo)] and dynamic [dynamic amplitude of low-frequency fluctuations (dALFF), dynamic regional homogeneity (dReHo)] metrics inter-group differences between pontine infarction (PI) and healthy controls (HCs) group.

Brain regions	Cluster size (voxels)	Peak intensity	MNI coordinates
**Brain regions with different sALFF between PI and HCs group**			
**LPI group**			
Left inferior temporal gyrus	60	4.8141	−51, −45, −15
Left caudate nucleus	70	4.7866	−9, 12, 18
Right caudate nucleus	71	5.0946	12, 9, 18
**RPI group**			
Right caudate nucleus	114	6.5688	18, 18, 9
Left superior occipital gyrus	44	-4.9682	−15, −87, 24
Left caudate nucleus	96	5.2077	−9, 21, 6
Right cuneus	57	-4.9775	9, −78, 24
**Brain regions with different dALFF between PI and HCs group**			
**LPI group**			
Left inferior temporal gyrus	74	4.412	−57, −48, −12
Right caudate nucleus	45	4.6241	9, 12, 18
**RPI group**			
Left caudate nucleus	70	4.9357	−15, 12, 21
Right caudate nucleus	109	4.6963	15, 15, 12
**Brain regions with different dReHo between PI and HCs group**			
**RPI group**			
Left middle cingulate cortex	72	-4.1225	−9, −39, 30

dALFF, dynamic amplitude of low-frequency fluctuations; dReHo, dynamic regional homogeneity; LPI, left pontine infarction; RPI, right pontine infarction; sALFF, static amplitude of low-frequency fluctuations; sReHo, static regional homogeneity.

Compared to the HCs group, the LPI group showed significantly increased sALFF in the left inferior temporal gyrus (ITG_L) and bilateral caudate nucleus (CN); the RPI group showed increased sALFF in the bilateral caudate nucleus (CN), and decreased sALFF in the right cuneus (CUN_R) and left superior occipital gyrus (SOG_L).

The LPI group displayed significantly increased dALFF in left inferior temporal gyrus (ITG_L) and right caudate nucleus (CN_R) when compared with HCs group. In the RPI group, the patients displayed increased dALFF in bilateral caudate nucleus (CN).

### Differences in sReHo and dReHo

Compared with the HCs group, there were no discernible alterations in sReHo between the HCs and PI groups with multiple comparison corrected (GRF, *P* < 0.05).

**FIGURE 3:**
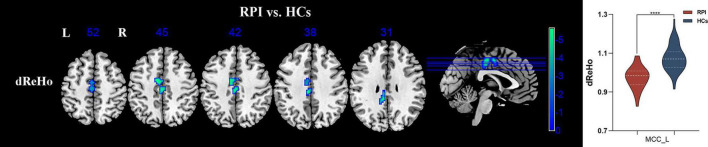
sReHo/dReHo differences between pontine infarction (PI) and healthy controls (HCs) group. Voxel-based analysis showed brain regions with significant dReHo alterations of RPI group. dReHo, dynamic regional homogeneity; RPI, right pontine infarction; sReHo, static regional homogeneity. ****Represents *p*-value < 0.0001.

The significant inter-group differences in dReHo were shown in Figure 3, Table 2. Compared with the HCs group, the RPI group displayed significantly decreased dReHo in left middle cingulate cortex (MCC_L). However, there were no discernible alterations in dReHo between the HCs and LPI groups with multiple comparison corrected (GRF, *P* < 0.05).

### Correlational analysis

**FIGURE 4:**
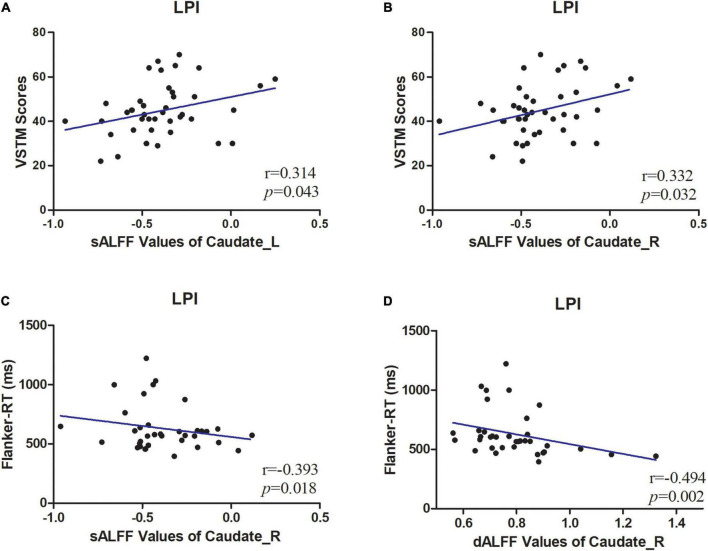
Correlations between sALFF/dALFF values and behavioral scores. **(A)** The patients in LPI group shown a significantly positive correlation between sALFF values of CN_L and verbal short-term memory (VSTM) scores. **(B)** The patients in LPI group shown a significantly positive correlation between sALFF values of CN_R values and VSTM scores. **(C)** The patients in LPI group shown a significantly negative correlation between sALFF values of CN_R and Flanker-RT scores. **(D)** The patients in LPI group shown a significantly negative correlation between dALFF values of CN_R and Flanker-RT scores. dALFF, dynamic amplitude of low-frequency fluctuations; LPI, left pontine infarction; RPI, right pontine infarction; sALFF, dynamic amplitude of low-frequency fluctuations.

To further explore the correlation between these metrics and cognitive function. The mean static metrics (sALFF) and dynamic metrics (dALFF, dReHo) values of the areas exhibiting differences in patients were extracted to perform the correlation analysis. Only LPI group evinces a significant correlation with behavioral scales (Figure 4). In detail, in LPI group, the increased sALFF in the CN_L were positively correlated with VSTM scores (*p* = 0.043, *r* = 0.314) (Figure 4A), and the increased sALFF in the CN_R was positively correlated with VSTM scores (*p* = 0.032, *r* = 0.332) (Figure 4B). the increased sALFF in the CN_R were negatively correlated with Flanker-RT scores (*p* = 0.018, *r* = −0.393) (Figure 4C), and the increased dALFF in the CN_R were negatively correlated with Flanker-RT scores (*p* = 0.002, *r* = −0.494) (Figure 4D). No discernible correlation was detected between the regions exhibiting inter-group distinctions and behavioral scales in the RPI group, nor between the VLTM scores and these metrics.

### Receiver operating characteristic (ROC) curve analysis

**FIGURE 5:**
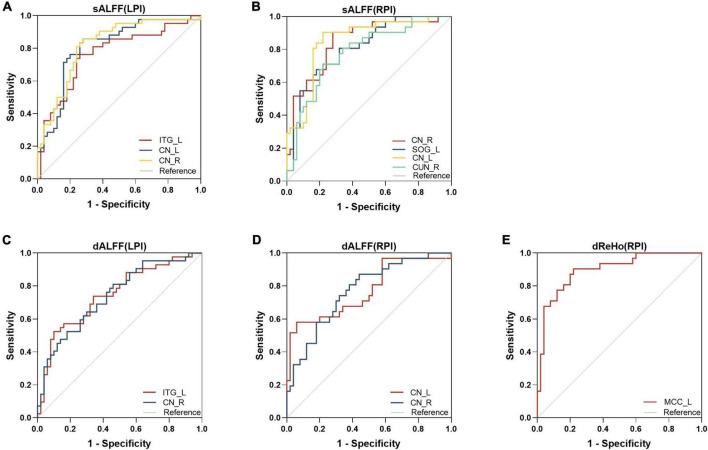
Receiver operating characteristic (ROC) analyses of sALFF **(A,B)**, dALFF **(C,D)**, and dReHo **(E)** in the defined region of interests (ROIs). dALFF, dynamic amplitude of low-frequency fluctuations; dReHo, dynamic regional homogeneity; LPI, left pontine infarction; RPI, right pontine infarction; sALFF, static amplitude of low-frequency fluctuations.

The ROC curves of altered static metrics and dynamic metrics were shown in Figure 5 and Supplementary Table 1. In the static analysis, the results demonstrated that the area under the curves (AUC) of the increased sALFF in ITG_L, CN_L, CN_R were 0.763, 0.810, 0.821, respectively (Figure 5A); the AUC of the increased sALFF in CN_R, CN_L and decreased sALFF in SOG_L, CUN_R were 0.845, 0.807, 0.852, 0.781, respectively (Figure 5B); the AUC of the increased dALFF in ITG_L, CN_R were 0.745, 0.739, respectively (Figure 5C); the AUC of the increased dALFF in CN_L, CN_R were 0.772, 0.769, respectively (Figure 5D); the AUC of the decreased dReHo in MCC_L was 0.898 (Figure 5E).

## Discussion

In the study, the patients with chronic pontine infarction showed aberrant static spontaneous brain activity and dynamic temporal variances with multiple metrics. The altered regions involved the supratentorial regions, including cortex and subcortical. These findings suggest that patients with unilateral subtentorial infarction may damage the direct or indirect anatomical connections between the brain stem and distant cortical and subcortical areas.

Compared with healthy controls, the LPI group displayed abnormal brain activity of sALFF/dALFF mainly in inferior temporal cortex and basal ganglia. The RPI group also showed activated sALFF/dALFF in the basal ganglia, in addition to decreased sALFF activity in visual cortex. Inferior temporal cortex is crucial for visual recognition and is considered to be the final stage in the ventral cortical visual system. The basal ganglia are not only mainly responsible for motor control (Bonelli and Cummings, 2007), but also for regulating cognitive and executive functions (Brown et al., 1997). In the study, the stroke patients displayed increased brain activities in the inferior temporal cortex and basal ganglia both in LPI and RPI groups, which was in line with the previous studies (Ni et al., 2016; Chen et al., 2018). These results may indicate that these activated regions were a potential compensation effect to motor and cognitive impairment in patients with pontine infarction. Furthermore, the activation in the inferior temporal gyrus may be linked to the recovery of motor function following a stroke, since it is necessary for visual guidance in the process (Archer et al., 2016). We also found that there was significantly correlation between the altered in sALFF/dALFF the basal ganglia and the short-term verbal memory, and visual memory. It may be to compensate for the motor and underlying cognitive impairment in the subtentorial infarction involving the motor pathways. This was in line with the previous finding that patients with unilateral basal ganglia lesions exhibit a decline in memory function (Ell et al., 2010). This may be attribute to the lesion disrupts the connection of the cortico-basal ganglia-cerebellar loop by way of the brainstem.

As well, we also found the decreased activity of static ALFF in the visual cortical in the RPI group. The visual cortex is a typical visual information system, mainly participating in the functional activities related to visual formation, visual perception, visual memory, and higher-level visual association processes (Grossberg et al., 1997; Khan et al., 2011). The decreased spontaneous brain activity in the visual cortex may reflect functional interruption of the cognitive perceptual system. Furthermore, the visual cortex is an integral part of cortical-pontine-cerebellar pathway in the anatomy, and pontine lesions may damage to corticopontine tract, which can lead to a disruption of the corresponding cortical function.

In the study, there was no cerebral regions which showed significant sReHo/dReHo differences in the LPI group relative to the HC group. The RPI patients exhibited decreased dReHo in the middle cingulate cortex. The results of the prior study were consistent with the finding that pontine infarction patients displayed decreased function connection in middle cingulate cortex (Chen et al., 2019). The cingulate cortex is usually considered to be a part of the limbic system (Rolls, 2019), which is associated with memory, sleep, emotions, and behavior. The previous study suggested that the middle cingulate cortex was strongly activated during both action observation and execution (Di Cesare et al., 2021). The decreased activity in the middle cingulate cortex may reflect the underlying basis for the impairment of executive function and cognition in patients with chronic pontine infarction. However, the mechanisms of these complex functional alterations should be determined by future studies.

This research investigated a variety of indicators, including static and dynamic, and these results revealed both heterogeneity and similarities, which emphasizes the significance of studying multiple indicators. As they can provide differing and complementary information about local aberrant brain activity, thus offering a more comprehensive insight into the characteristics of functional impairment and recovery in patients with subtentorial infarction. Furthermore, the ROC analysis of a variety of metrics revealed that local brain activity had the potential capacity to distinguish subtentorial infarction patients with functional deficits from healthy controls, and suggested the stability of these results. In addition, the results of this study also exhibited significant heterogeneities between the LPI and RPI groups when compared with the normal controls, which was in line with our previous, revealing a lesion-side effect. This may be attributed to the fact that right-handed patients with a left hemisphere infarction have more difficulty with daily activities than those with a lesion in the right hemisphere, thus reducing the practice of daily activities conducive to recovery. However, the complex mechanisms of lesion-side effect in subtentorial infarction should be determined in future studies.

## Limitation

There were some limitations to this experiment. Firstly, the sample size was limited due to the stringent inclusion criteria, thus it is necessary to increase the sample size to further validate this result in future studies. Secondly, there were significant inter-group differences in sex in the LPI group, future investigations should expand the sample size to balance the differences in sex. Thirdly, the modified Rankin Scale will be included as a crucial evaluation scale to evaluate the chronic functional status of the patients in the future researches. Finally, this study was conducted in a single time frame and did not explore longitudinal changes from the acute to the chronic phase. A longitudinal research should be carried out to investigate the dynamic changes in local brain activity over time.

## Conclusion

In conclusion, there were aberrant local brain activity and temporal dynamic variances with multiple metrics in patients with unilateral pontine infarction involving motor pathways. The subtentorial infarction-induced cerebral activation changes both in the motor and cognitive systems, suggesting the imbalances between functional damage and reorganization at the whole-brain level in these patients. This may indicate that there is a reciprocal effect between motor and cognitive impairment and repair.

## Data availability statement

The raw data supporting the conclusions of this article will be made available by the authors, without undue reservation.

## Ethics statement

The studies involving human participants were reviewed and approved by the First Affiliated Hospital of Zhengzhou University, Tianjin Medical University General Hospital and Tianjin Huanhu Hospital. The participants provided their written informed consent to participate in this study.

## Author contributions

XW, CW, and CR conceived and designed the experiment. XW and CW drafted and revised the manuscript. XW, CW, and YW analyzed the imaging data. JLiu, JG, PM, YW, and YYW collected the original data, including perform MRI scans and behavioral assessments. ZL, JLi, KW, YZ, and JC provided guidance and advice. All authors contributed to the article and approved the submitted version.

## Footnotes

1
https://pstnet.com/products/e-prime/

2
http://rfmri.org/dpabi
