# Supplementary material for: Altered static and dynamic spontaneous neural activity in patients with ischemic pontine stroke
Source: Front Neurosci. 2023 Mar 16;17:1131062. doi: 10.3389/fnins.2023.1131062 (PMC10060846; doi:10.3389/fnins.2023.1131062)
Supplement: Supplementary file 1 [file Data_Sheet_1.doc]

***Supplementary Material***

**Altered static and dynamic** **spontaneous neural activity in patients with ischemic** **pontine stroke**

Xin Wang^1†^, Caihong Wang^1*†^, Jingchun Liu^2^, Jun Guo^3^, Peifang Miao^1^, Ying Wei^1^, Yingying Wang^1^, Zhen Li^4^, Jie Li^4^, Kaiyu Wang^5^, Yong Zhang^1^, Jingliang Cheng^1^, Cuiping Ren^*^

^*^**Correspondence:** Caihong Wang, No.1 Jianshe Dong Road, Erqi district, Zhengzhou 450052, China. Emails: [fccwangch@zzu.edu.cn;](mailto:fccwangch@zzu.edu.cn:) Cuiping Ren, No.1 Jianshe Dong Road, Erqi district, Zhengzhou 450052, China. Emails: [rcp810@sohu.com.](mailto:fccwangch@zzu.edu.cn:)


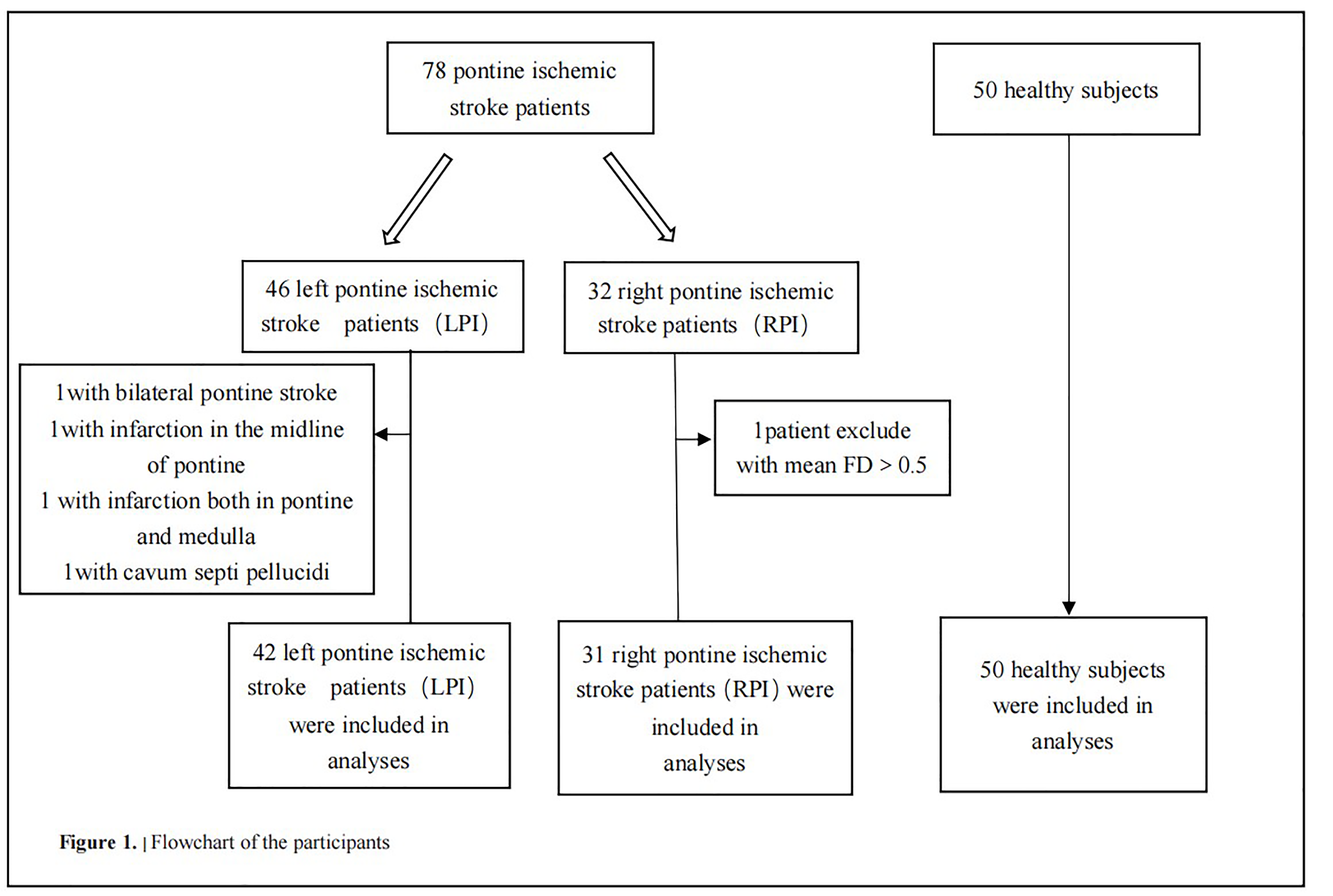


**Figure 1.** Flowchart of the participants

| **Table 1.** Receiver operating characteristic (ROC) analyses of brain regions showing inter-group differences between PI and NC group | | | |  |
| --- | --- | --- | --- | --- |
| **Regions** | **Side** | **AUC** | ***p* value** |  |
| **ROC analyses of sALFF in the definend ROIs** | | | |  |
| **LPI group** |  |  |  |  |
| inferior temporal gyrus | L | 0.763 | < 0.0001* |  |
| caudate nucleus | L | 0.810 | < 0.0001* |  |
| caudate nucleus | R | 0.821 | < 0.0001* |  |
| **RPI group** |  |  |  |  |
| caudate nucleus | R | 0.845 | < 0.0001* |  |
| superior occipital gyrus | L | 0.807 |  |  |
| caudate nucleus | L | 0.852 | < 0.0001* |  |
| cuneus | R | 0.781 | < 0.0001* |  |
| **ROC analyses of dALFF in the definend ROIs** | | | |  |
| **LPI group** |  |  |  |  |
| inferior temporal gyrus | L | 0.745 | < 0.0001* |  |
| caudate nucleus | R | 0.739 | < 0.0001* |  |
| **RPI group** |  |  |  |  |
| caudate nucleus | L | 0.772 | < 0.0001* |  |
| caudate nucleus | R | 0.769 | < 0.0001* |  |
| **ROC analyses of dReHo in the definend ROIs** | | | |  |
| **RPI group** |  |  |  |  |
| middle cingulate cortex | L | 0.898 | < 0.0001* |  |
| *Significantly difference between PI patients and HCs in Area Under Curve (AUC) (*p*<0.05) | | | |  |

Abbreviations: dALFF, dynamic amplitude of low-frequency fluctuations; dReHo, dynamic regional homogeneity; LPI, left pontine infarction; RPI, right pontine infarction; sALFF, static amplitude of low-frequency fluctuations.


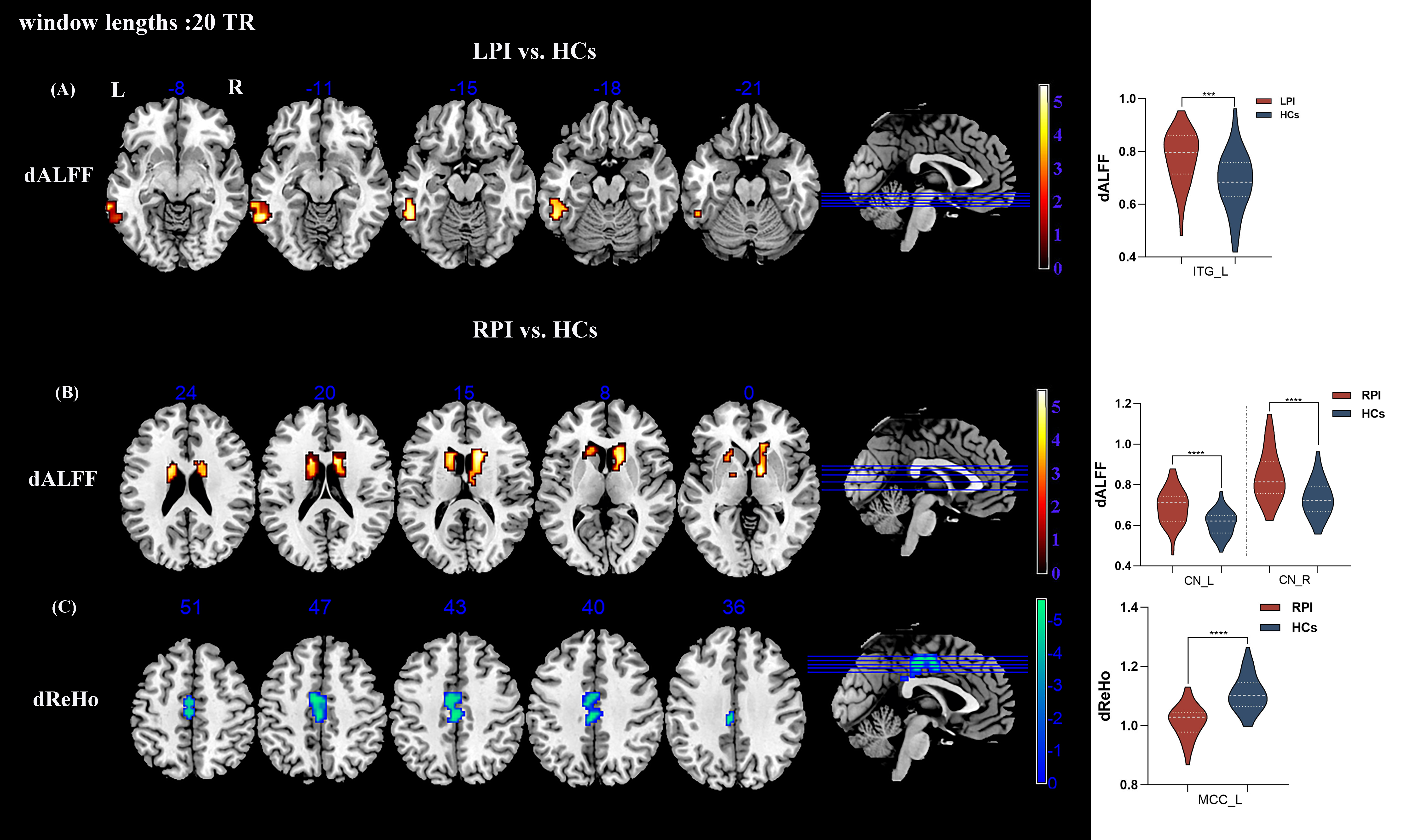


**Figure 2. dALFF/dReHo differences between PI and NC groups. (window lengths :20 TR).** (A)Voxel-based analysis showed brain regions with significant dALFF alterations of LPI group. (B)Voxel-based analysis showed brain regions with significant dALFF alterations of RPI group. (C)Voxel-based analysis showed brain regions with significant dReHo alterations of RPI group.

Abbreviations: CN_L, left caudate nucleus; CN_R, right caudate nucleus; ITG_L, left inferior temporal gyrus; LPI, left pontine infarction; MCC_L, left middle cingulate cortex; RPI, right pontine infarction; ***Represents *p* value = .0001; ****Represents *p* value < .0001.

| **Table 2.** Dynamic (dALFF, dReHo) metrics inter-group differences between PI and NC group(window lengths :20 TR） | | | |
| --- | --- | --- | --- |
| **Brain regions** | **Cluster size(voxels)** | **Peak intensity** | **MNI coordinates** |
| **Brain regions with different dALFF between PI and NC group** | | | |
| **LPI group** |  |  |  |
| left inferior temporal gyrus | 75 | 4.2166 | -57 -48 -12 |
| **RPI group** |  |  |  |
| left caudate nucleus | 85 | 5.2297 | -15,15,18 |
| right caudate nucleus | 133 | 4.7875 | 15,15,12 |
| **Brain regions with different dReHo between PI and NC group** | | | |
| **RPI group** |  |  |  |
| left middle cingulate cortex | 94 | -4.2843 | -6, -36,30 |

Abbreviations: dALFF, dynamic amplitude of low-frequency fluctuations; dReHo, dynamic regional homogeneity; LPI, left pontine infarction; RPI, right pontine infarction


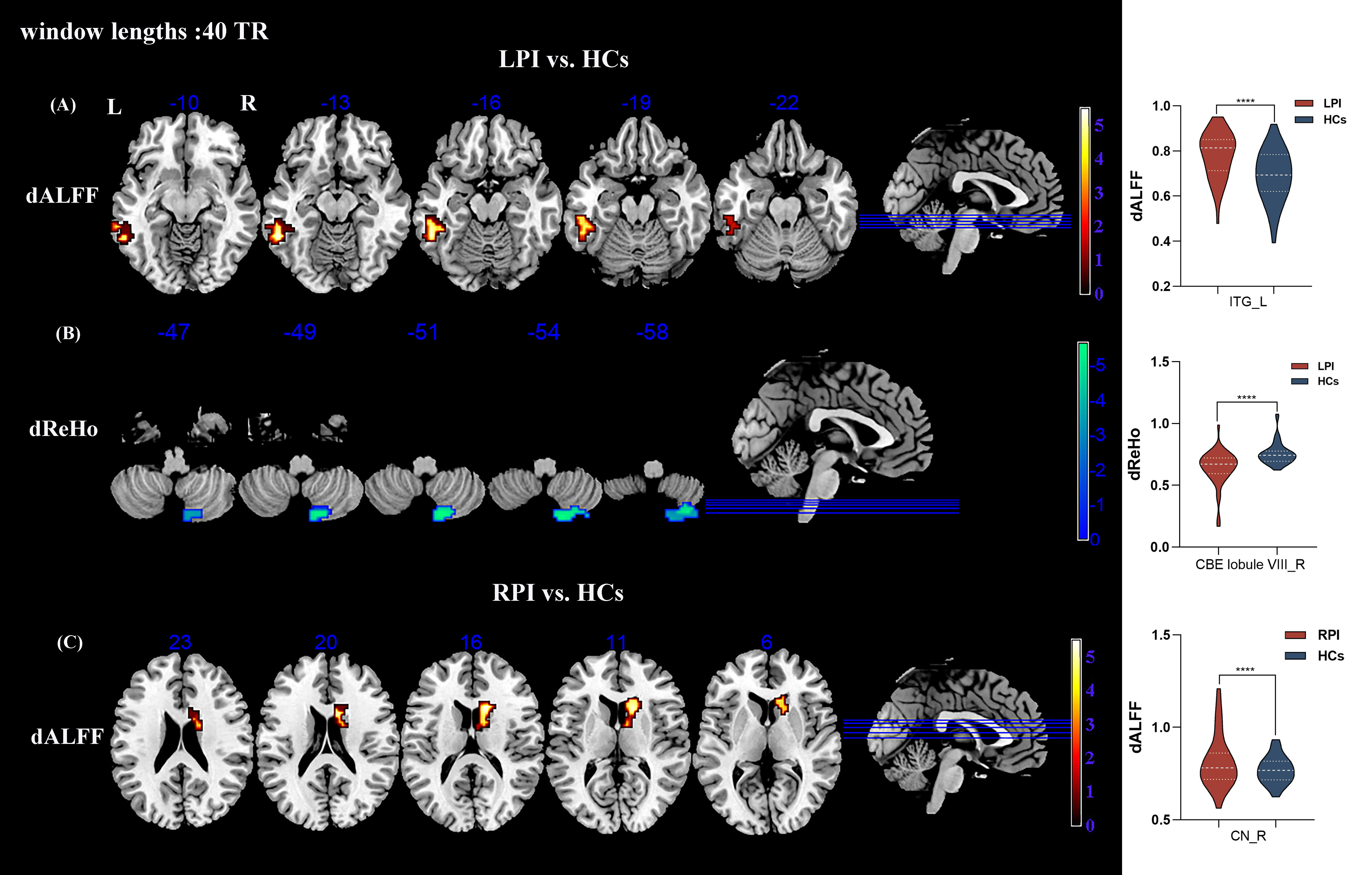


**Figure 3.** **dALFF/dReHo differences between PI and NC groups. (window lengths :40 TR).** (A)Voxel-based analysis showed brain regions with significant dALFF alterations of LPI group. (B)Voxel-based analysis showed brain regions with significant dReHo alterations of LPI group. (C)Voxel-based analysis showed brain regions with significant dALFF alterations of RPI group.

Abbreviations: CBE lobule VIII_R, right cerebellar lobule VIII; CN_R, right caudate nucleus; ITG_L, left inferior temporal gyrus; LPI, left pontine infarction; RPI, right pontine infarction; ****Represents *p* value < .0001.

| **Table 3.** Dynamic (dALFF, dReHo) metrics inter-group differences between PI and NC group (window lengths :40 TR） | | | | | | | |
| --- | --- | --- | --- | --- | --- | --- | --- |
| **Brain regions** | | **Cluster size(voxels)** | **Peak intensity** | | | | **MNI coordinates** |
| **Brain regions with different dALFF between PI and NC group** | | | | | | | |
| **LPI group** | |  |  | |  | | |
| left inferior temporal gyrus | | 75 | 4.2166 | | | | -57,-48,-12 |
| **RPI group** | |  |  | | | |  |
| right caudate nucleus | | 80 | 4.5168 | | | | 12,15,18 |
| **Brain regions with different dReHo between PI and NC group** | | | | | | | |
| **LPI group** |  | | |  | |  | |
| right cerebellar lobule VIII | 21 | | | -4.0165 | | 15, -78, -51 | |

Abbreviations: dALFF, dynamic amplitude of low-frequency fluctuations; dReHo, dynamic regional homogeneity; LPI, left pontine infarction; RPI, right pontine infarction
